# Superfast assembly and synthesis of gold nanostructures using nanosecond low-temperature compression via magnetic pulsed power

**DOI:** 10.1038/ncomms14778

**Published:** 2017-03-16

**Authors:** Binsong Li, Kaifu Bian, J. Matthew D. Lane, K. Michael Salerno, Gary S. Grest, Tommy Ao, Randy Hickman, Jack Wise, Zhongwu Wang, Hongyou Fan

**Affiliations:** 1 Sandia National Laboratories, Albuquerque, New Mexico 87185, USA; 2 Cornell High Energy Synchrotron Source, Cornell University, Ithaca, New York 14853, USA; 3 Department of Chemical and Biological Engineering, Center for Micro-Engineered Materials, University of New Mexico, Albuquerque, New Mexico 87106, USA

## Abstract

Gold nanostructured materials exhibit important size- and shape-dependent properties that enable a wide variety of applications in photocatalysis, nanoelectronics and phototherapy. Here we show the use of superfast dynamic compression to synthesize extended gold nanostructures, such as nanorods, nanowires and nanosheets, with nanosecond coalescence times. Using a pulsed power generator, we ramp compress spherical gold nanoparticle arrays to pressures of tens of GPa, demonstrating pressure-driven assembly beyond the quasi-static regime of the diamond anvil cell. Our dynamic magnetic ramp compression approach produces smooth, shockless (that is, isentropic) one-dimensional loading with low-temperature states suitable for nanostructure synthesis. Transmission electron microscopy clearly establishes that various gold architectures are formed through compressive mesoscale coalescences of spherical gold nanoparticles, which is further confirmed by *in-situ* synchrotron X-ray studies and large-scale simulation. This nanofabrication approach applies magnetically driven uniaxial ramp compression to mimic established embossing and imprinting processes, but at ultra-short (nanosecond) timescales.

## Abstract (web summary)

Gold nanostructures have shape-dependent properties, making synthetic control over their morphology critical. Here, the authors use dynamic compression to obtain a variety of gold nanoarchitectures, which are formed at very fast timescales by the controlled coalescence of spherical particle arrays.

Gold nanostructures such as nanoparticles, nanorods, nanowires and nanosheets are promising building blocks for important applications in biosensing, catalysis, imaging and therapeutics^1^,^2^,^3^,^4^,^5^,^6^,^7^,^8^,^9^. An ability to tune the size and shape of the gold nanostructures beyond the nanometre scale is essential for such applications. It has prompted extensive efforts to develop synthetic methods and engineering processes to manipulate gold nanostructures. Among previous efforts, chemical methods have been the dominant method to synthesize a variety of nanostructures. For example, redox reactions have been conducted in solution phases using gold precursors and reductants to induce seed formation, nucleation and crystal growth. Based on the minimum free energy of material systems, different sizes and shapes such as nanoparticles, nanorods, nanowires and nanosheets are achieved through crystal growth. Through chemical methods, several strategies have been developed to provide fine control of size and shape. For example, organic ligands such as alkanethiols have been used to bind to the nanoparticle surface, preventing nanoparticle aggregation and maintaining monodisperse spherical particles^10^. Capping agents such as poly(vinyl pyrrolidone) (PVP) have also been used to inhibit certain facet growth of seed crystals for orientated growth of nanorods and nanowires^11^.

The so-called ‘galvanic replacement' method was developed in a mild etching process using nanocubes as sacrificial templates to synthesize hollow gold and silver nanostructures^9^,^12^. Galvanic replacement has been further modified to synthesize even more complex nanostructures and/or compositions by coupling with other processes such as co-reduction and the Kirkendall effect^2^,^3^,^13^. Templating method is another effective means recently developed to confine fast reaction kinetics, forming well-defined one-dimensional chain-like structures^14^,^15^. However, due to the fast kinetics associated with chemical reactions, it has been fundamentally limited for chemical synthesis methods only to engineer or control resultant nanomaterial structures.

Recently, it has been shown that static pressure can induce phase transition of nanocrystals. Essentially static pressure alters defect motions or arrangement of atoms in particle atomic lattices through phase transformation^16^,^17^,^18^,^19^,^20^,^21^. Depending on the pressure magnitude, different phase and structural transformations have been observed. Under applied pressures up to 10 GPa, assemblies of gold and silver nanoparticles with sizes of 5–10 nm undergo a reversible compression^18^,^19^. At even higher pressure, disordered microstructure can be rearranged through phase transformation to achieve much better mechanical performance^18^,^19^. Detailed transmission electron microscopy (TEM) investigations in atomic lattice rearrangement and twinning confirmed these phase transformations^18^,^19^. These studies clearly establish that static pressure can effectively create new atomic modes of microstructures, which relax surface and internal strains induced by the stress.

Here we demonstrate the use of superfast dynamic compression generated using magnetically driven isentropic loading to assemble and fabricate robust gold nanostructures. The compressive synthesis is accomplished through controlled coalescence of organic ligand-coated gold nanoparticles within nanosecond compression times. The uniaxial strain produced by one-dimensional ramp waves compresses and shears the ordered nanoparticle superlattice, forcing the nanoparticles to coalesce into one- to three-dimensional gold architectures including nanorods, nanowires and nanosheets, depending on the orientation of the nanoparticle superlattice to the propagating ramp wave.

## Results

### Gold nanoparticle arrays

**Figure 1:**
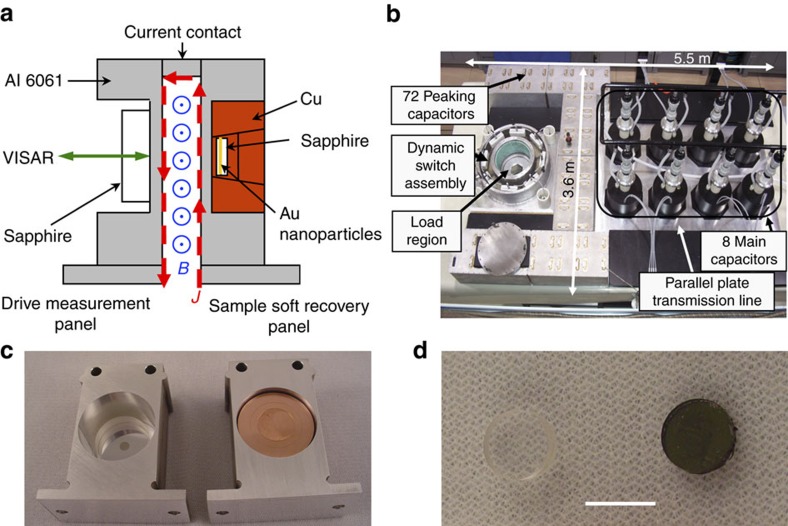
Magnetically driven dynamic compression of nanocrystals. (**a**) A schematic cross-section of the load panels, (**b**) the Veloce pulsed power generator, (**c**) drive measurement panel (left) and sample soft recovery panel (right) and (**d**) sapphire substrate (left) and gold nanocrystal arrays coated sapphire substrate (right). Scale bar in **d** is 1 cm.

Self-assembled arrays of organic ligand-coated spherical gold nanoparticles were created as initial materials for dynamic compression. The gold nanoparticles were synthesized using a one-phase method to produce monodisperse nanoparticles with 5.2 nm diameter and s.d. of 4.2% (ref. 22). Then a solution of these nanoparticles was prepared by combining 80 mg of 5.2 nm gold nanoparticles, and 20 mg of polystyrene in 1 ml of toluene solvent (see Methods). Thin films of ordered gold nanoparticle arrays were fabricated through spin coating of the gold nanoparticle solution on sapphire wafers at a spinning speed of 1,500 r.p.m. The initial state of the film was characterized using scanning tunnelling microscopy, transmission electron microscopy (TEM) and X-ray diffraction (XRD). Supplementary Fig. 1 illustrates the structure of the initial self-assembled nanoparticle film. The XRD spectrum, in Supplementary Fig. 1a, was shown to fit a face-centred cubic (fcc) superlattice using Fit2D software. The unit cell parameter *a* was calculated to be 10.4 nm. Figure 1b shows a typical scanning electron microscopy (SEM) image of the gold nanoparticle arrays. Fast-Fourier transformation (FFT) analysis (inset in Supplementary Fig. 1b) further confirms that the periodic structure has an fcc symmetry in the *Fm-*3*m* space group.

### Magnetic dynamic compression and sample recovery

**Figure 2:**
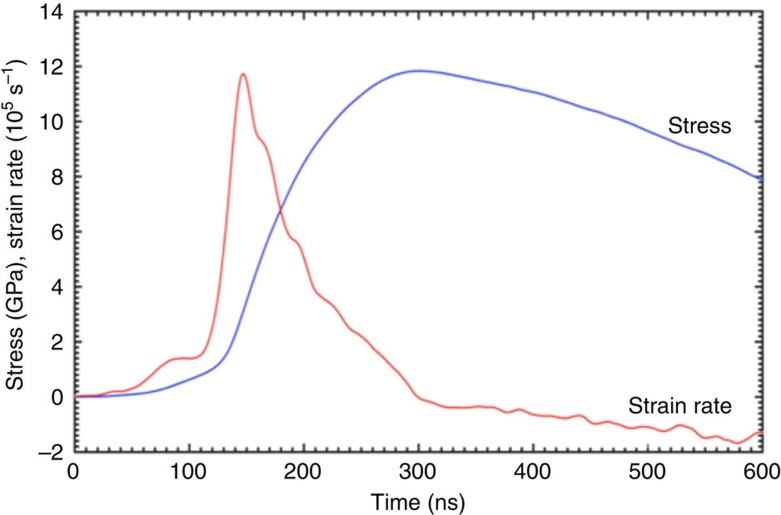
Dynamic magnetic pressure and strain rate for the compressive synthesis. The pressure and strain rate are calculated from back-surface velocimetry interferometer system for any reflector measurements (VISAR) from Veloce^35^. Here the stress wave is configured to reach a peak stress of ∼12 GPa without producing a shock front within the samples. The dynamic peak pressure is maintained for only a few nanoseconds before the stress drops. A soft capture mechanism is critical to preserve the synthesized gold nanostructures.

Sandia's Veloce pulsed power machine is shown schematically in Fig. 1 (ref. 23), as configured for a magnetic-drive experiment. Veloce is a scaled down version of the Z-machine, a pulsed-power device which Sandia has pioneered^24^. Both machines were specifically designed for studying the response of materials under extreme conditions of pressure, temperature and radiation. Veloce is a medium-voltage, high-current, compact pulsed-power generator based on a strip-line design. The machine delivers up to 3 MA of current rapidly over about 400 ns into two inductive current carrying plates, where extreme magnetic forces are produced. As shown in Fig. 1a, the current density 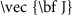 flows up one plate and down the other, producing an out-of-plane magnetic field 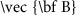 between them. The 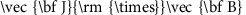 Lorentz force drives the two plates and mounted samples apart, accelerating each to 100 s of m s^−1^ over just a few nanoseconds. The machine can be configured to drive abrupt shocks, but in these experiments the plates and current pulse are shaped to produce a smooth shockless mechanical stress wave. This magnetic loading technique allows planar, continuous compression of materials several hundred microns in thickness and tens of millimetres in diameter to stresses approaching 15 GPa and strain rates up to 10^6^ s^−1^. Figure 1b shows the Veloce pulsed power generator for the compression of gold nanoparticle arrays. Figure 1c shows the capture capsule that was designed to allow soft recovery of nanoparticle samples for structural studies^25^. The thin film of gold nanoparticle arrays (Fig. 1d) on the sapphire substrate is directly used for compression synthesis. An example loading profile is shown in Fig. 2. In the shockless configuration (that is, isentropic or ramp wave), Veloce can reach these high pressures without significant heating of the sample.

### TEM of gold nanostructures from dynamic compression

**Figure 3:**
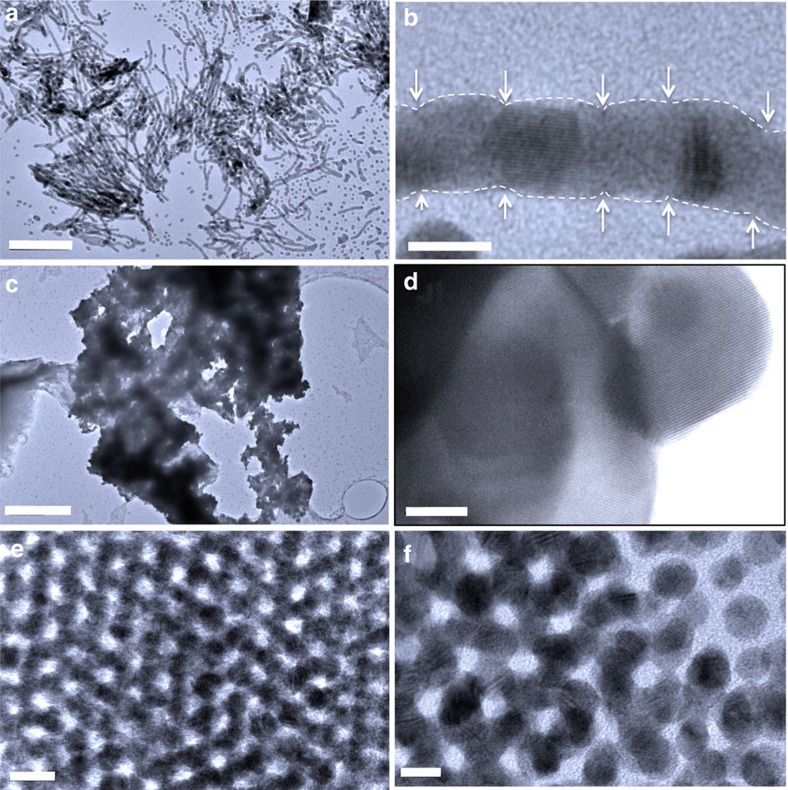
TEM images of consolidated gold nanostructures. (**a**) Representative TEM image of the gold nanowires. (**b**) HRTEM image of a gold nanowire. The white dashed lines indicate the profile of individual connected gold nanocrystals. Arrows point to the interface where the gold nanocrystal coalesce. (**c**) Representative TEM image of gold nanosheets. (**d**) HRTEM image of a nanosheet with an fcc lattice fringe of (111). (**e**) Representative TEM image of three-dimensional gold networks. (**f**) HR TEM image of the three-dimensional networks showing the edge with three dimensionally connected nanocrystals. Scale bars: (**a**) 100 nm; (**b**) 5 nm; (**c**) 400 nm; (**d**) 10 nm; (**e**) 10 nm; (**f**) 5 nm.

Experiments were conducted with increasing peak stresses of up to 12 GPa . Below 7 GPa, no nanostructural consolidation was observed in recovered samples. Between 7 and 12 GPa, significant coalesced nanostructures were dynamically fabricated through uniaxial compression. TEM images of the resultant gold nanostructures are shown in Fig. 3. One-dimensional nanowires, two-dimensional nanosheets and three-dimensionally connected networks were observed. For one-dimensional nanostructures, hexagonal arrays (or bundles) of long nanowires up to 200 nm were observed (Fig. 3a and Supplementary Fig. 2). The bundles can be dissolved in organic solvents such as toluene to obtain homogenous solution of nanowires. High-resolution TEM (HRTEM) imaging confirms gold nanowires consist of individual gold nanocrystals that are coalesced along the nanowire *c* axis. The nanowires are polycrystalline and twisted (Fig. 3a,b). From lattice spacing measurements, we found that the nanocrystal coalescence follows a random pattern. Only fcc (111) and (200) lattice fringes were found at these coalesced nanocrystals indicating they connect through (111) or (200) faces. HRTEM images (Fig. 3 and Supplementary Figs 2 and 3) further reveal that short gold nanorods are formed from a few (2–8) gold nanoparticles. The connections between two original nanoparticles where coalescence occurs are normally narrower, forming a neck configuration, which makes it easier to locate the original nanoparticles and their interfaces. As two-dimensional nanostructure, layers of gold nanosheets were found in clusters of several microns (Fig. 3c). HRTEM of the edge (Fig. 3d) confirms that these large clusters were composed with tens micron size single crystal gold nanosheets. The measured thickness of the gold sheets is about 12–24 nm (Supplementary Fig. 3). For three-dimensional gold architectures (Fig. 3e,f), several distinguished features are observed. First, the specimens remain ordered after compression up to ∼12 GPa. The TEM image confirms the ordered nature in the final gold network. Second, as shown in the TEM images (Fig. 3f and Supplementary Fig. 4), the nanostructures are three-dimensional interconnected. As a result, a nanoporous skeleton is formed with pore size ∼5 nm or smaller; the compression process causes the diameter of gold skeleton (5.8±0.5 nm) to become slightly larger than the diameter of the original spherical gold nanoparticles (5.1±0.3 nm). Third, branched gold framework (or skeleton) is formed by the multidirectional coalescence of spherical gold nanoparticles during dynamic compression (Supplementary Fig. 4).

### Structural evolution of gold nanoparticle arrays under stress

**Figure 4:**
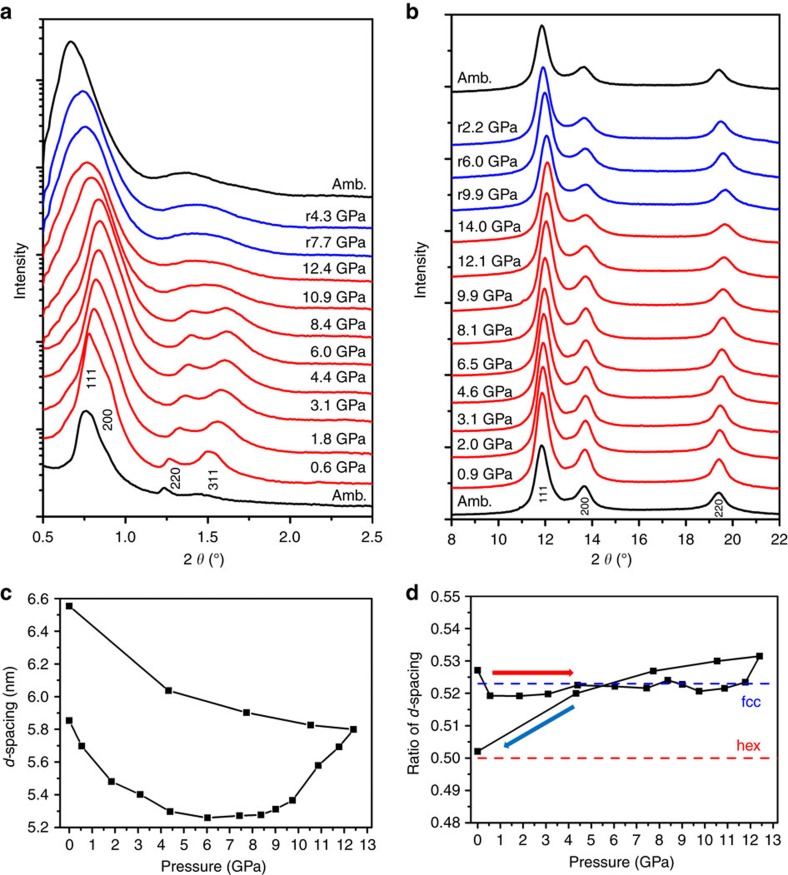
*In-situ* high-pressure synchrotron X-ray scattering patterns of gold nanoparticle arrays. (**a**) SAXS. (**b**) WAXS. (**c**) The *d*-spacings change along with the pressure during compression and release. The *d*-spacing of the first Bragg reflection in each high-pressure SAXS patterns is used. (**d**) *d*-spacing ratios (*R*) at different pressures. When the pressure is lower than ∼10 GPa, the nanocrystal assembly has fcc structure and *R*=*d*_311_/*d*_111_, where *R* is almost constant and is very close to the theoretical *d*_311_/*d*_111_=for fcc structure. At the intermediate stage, *R* varied between 0.522 and 0.5. After 12.4 GPa, *R* shows a linear correlation of *d*_1_/*d*_2_/*d*_3_=1/2/3, indicating a lamellar or two-dimensional hexagonal mesophases. For two-dimensional hexagonal mesophase *R* is ∼0.5, the theoretical ratio *d*_200_/*d*_100_, which is consistent with SAXS indexing.

To understand the formation process of gold nanostructures, we performed detailed *in-situ* synchrotron X-ray characterizations on the gold nanoparticle arrays within diamond anvil cells and separately explored the process using fully atomistic molecular dynamics simulations. In both cases we were able to reproduce an observed threshold pressure to the coalescence process. Although these detailed *in-situ* X-ray measurements were conducted under quasi-static condition, the simulations were fully dynamic compression. In the controlled environment of the diamond-anvil, pressures can be held arbitrarily long rather than applied over nanoseconds. We were able to use *in-situ* small (SAXS) and wide (WAXS) angle synchrotron X-ray scattering to directly observe the structural evolution as a function of compressive pressure for both the atomic lattice of the gold and the nanoparticle superlattice. *In-situ* high-pressure SAXS patterns (Fig. 4a) indicate that, upon continuous quasi-static increase of pressure, the observed peaks shift to higher angle, indicative of a pressure-induced shrinkage of the interparticle distance. This is further confirmed by the *d* spacing at varied pressures (Fig. 4c). At a threshold pressure of ∼9 GPa, several groups of nearby SAXS peaks start to merge; at 12.4 GPa, the number of peaks is reduced to 3. Three remaining peaks display a linear correlation of *d*_1_/*d*_2_/*d*_3_=1/2/3, indicating the formation of a lamellar or two-dimensional hexagonal mesophase. This mesophase transformation is consistent with results of *d* spacing ratio at different pressure (Fig. 4d) and also the formation of layered nanosheets and hexagonal bundles of gold nanowires from TEM imaging. Upon release of pressure to ambient condition, the mesophase was well preserved. *In-situ* high-pressure WAXS patterns (Fig. 4b) indicate that the overall atomic lattice shrinks upon applied pressure and returns to the original face center cubic phase.

### Molecular dynamics simulations

**Figure 5:**
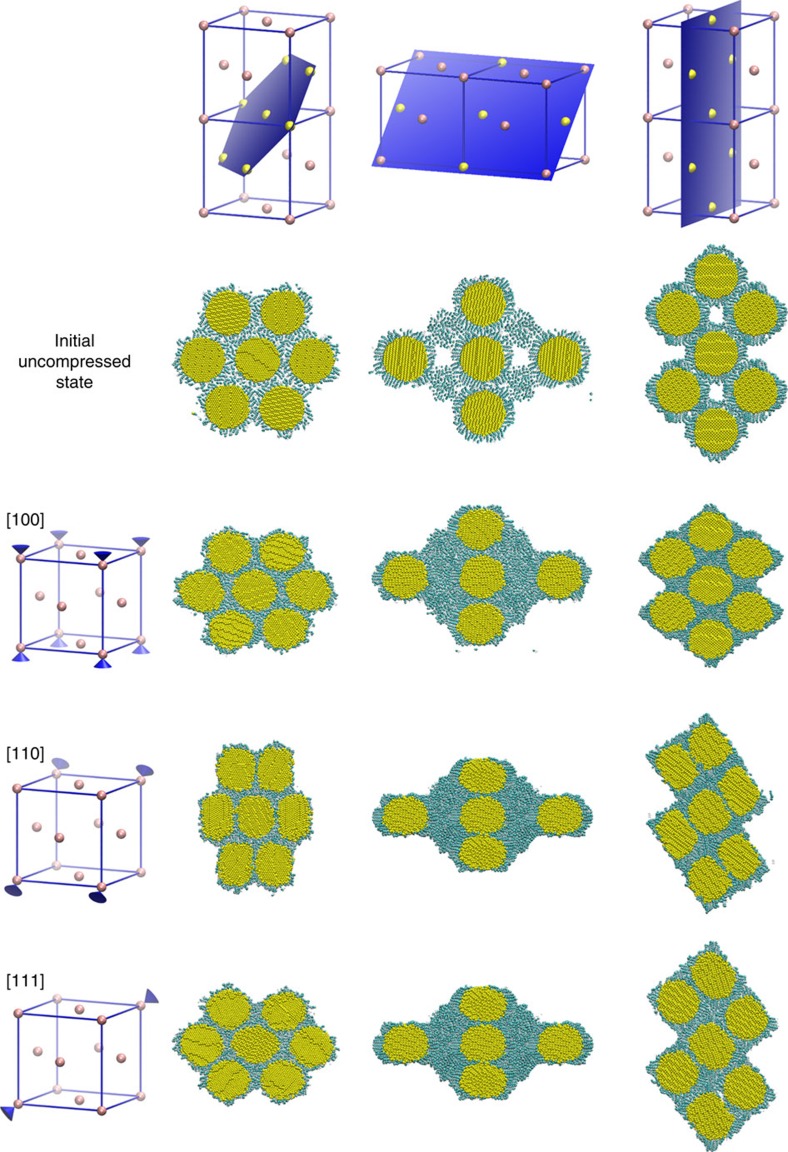
Atomistic simulations of an fcc superlattice of gold nanoparticles and their ligand coatings before and after dynamic compression. These illustrations in each row show nearest-neighbour cluster within the fcc lattice from along [100], [110] and [111] orientations after compression and initial uncompressed state. The clusters are cut along three lattice planes (columns) to show the three-dimensional nature of the resulting structures.

Classical molecular dynamics simulations were performed under high-rate compression. Nanoparticle superlattices were uniaxially compressed over hundreds of picoseconds to pressures comparable to the dynamic ramp experiments. Figure 5 shows slices of representative nearest-neighbour clusters for the initial and final configurations of the superlattice strained along the [100], [110] and [111] directions of the fcc lattice. The nearest-neighbour cluster representation shows how the 12 neighbours respond to compression along different orientations. While the initial fcc structure had the standard 12 nearest neighbours, compression along [110], [111] and [100], resulted in 2, 6 and 8 nearest neighbours, respectively. Both a nearest-neighbour analysis and the images illustrate the formation of wires in the [110] case. The [111] analysis is consistent with formation of three-dimensional, or possibly planar structures—since having six nearest neighbours is consistent with an in-plane hexagonal structure. The compression along [100] appears from the images to give three-dimensional structures, but the nearest-neighbour number of 8 indicates a three-dimensional structure with less symmetry than a perfect fcc lattice. During and following the compression, ligands are pressed and sheared away from the sintering points between the nanoparticle cores. The chains have been observed to both compact on the remaining particle surface, and to be pulled free of the surface on the timescales of the simulations. On longer timescales, and as pressure drops, we would expect that significant numbers of ligand chains would be released.

## Discussion

The combined results from TEM imaging, molecular simulations and synchrotron X-ray scattering unambiguously confirm the formation of several gold nanostructures via stress-induced coalescence of spherical gold nanocrystals. The applied magnetic stress effectively squeezes the nanocrystal lattice to shrink interparticle separation. This is confirmed by the X-ray results (Fig. 4), and the effect on nanostructures is determined by molecular modelling. This is confirmed by the individual nanocrystal domains observed within the final gold nanostructures (Fig. 3 and Supplementary Figs 2 and 4). Different final nanostructural geometries, produced here, are thought to be due to differently oriented superlattice domains in the initial polycrystalline nanoparticle film. Molecular dynamics confirmed that the nanostructure depends on the alignment between superlattice orientation and loading direction. Compression along the [110] orientation led to wire formation, while compression along [111] led to a three-dimensional nanostructure. Compression along [100] led to either planar aggregation or three-dimensional structures. If the nanofabrication were attempted through a highly dissipative shock compression process, the associated large temperature rise would be a critical concern due to heat-induced sintering. Static compression techniques, typically with diamond anvil cells, are used to examine a material's principal isotherm, which represents the pressure–volume response obtained by compressing a material from ambient conditions at constant temperature. However, shock compression is unsuitable for synthesis of nanostructures since the evaluated temperatures of shock states (typically >1,300 K) would induce melting of the nanoparticles. Temperatures during ramp compression (∼400 K) are substantially lower than the principal Hugoniot, which facilitates dynamic compression of nanoparticles into new nanostructures without thermally induced breakdown. The observation that the diameters of the gold nanocrystals remain unchanged unambiguously supports that the overall coalescence is induced by mechanic compression, not heat. The overall dynamic compression exhibits several advantageous features in synthesis of new nanostructure materials: the process is superfast and can fabricate materials within a few nanoseconds; because of the short-time compression, the nanocrystals might not have time for rotation, which leads to the formation of polycrystalline framework; and the compression process can be accomplished in uniaxial pressure field without requiring hydrostatic pressure field. Finally, the resultant nanostructures consist of interconnected nanocrystals as their main skeletons, which potentially provide enhanced mechanical stability according to the recent TEM results^18^,^19^. We expect that a wide range of building blocks, such as metallic (for example, Pd, Pt, Ag and so on), semiconductive (for example, CdSe) and magnetic (for example, FePt) nanoparticles can be used to fabricate new nanostructured materials by this method. Overall, the magnetically driven dynamic compression has proven to be quite successful and suitable for the fabrication of new nanostructures. Moreover, the experimental results demonstrated that the mechanical compression process is viable for high throughput and high-fidelity manufacture because of the high strain rate similar to embossing or imprinting processes.

## Methods

### Sample preparation

Spherical gold nanoparticles were synthesized based on the method described in the literature^22^. The resulting gold nanoparticles are 5.2 nm in diameter with a s.d. of 4.2%. Solutions of 5 nm Au nanoparticles were prepared by adding 80 mg of 5.2 nm gold nanoparticles and 20 mg of polystyerene with a molecular weight of 2 M g mol^−1^ in 1 ml of toluene. Thin films of ordered gold nanoparticle arrays were fabricated through casting or spin coating of the gold nanoparticle solutions on sapphire wafers at spinning speed of 1,500 r.p.m.

### Characterizations

TEM was performed on a JEOL 2010 with a 200 kV acceleration voltage, equipped with a Gatan slow scan CCD camera. SEM images were taken using a Hitachi S-5200 FEG microscope at 5 kV. *In-situ* HP-SAXS and HP-WAXS measurements were performed on B1 station at Cornell High Energy Synchrotron Source (CHESS) with monochromatic X-ray radiation of wavelength *λ* of 0.485946 Å (ref. 26). The distance between the sample and a large area MAR345 detector was 727.19 mm, as determined using both CeO_2_ and silver behenate powder standard. Several small ruby chips were loaded into DAC chamber as inside pressure probes to monitor the sample pressure by a standard pressure-dependent ruby fluorescence technique^27^. Scattering images were calibrated and integrated using the Fit2D software.

### Simulation methods

Classical molecular dynamics simulations of high-rate compression were performed using LAMMPS^28^ with an all-atom model of gold nanoparticles coated with dodecanethiol ligands. Hydrocarbon interactions were modelled using the OPLS force field^29^ modified with an updated dihedral potential^30^ and with nonbonded interactions using an exponential-6 form^31^. Gold atoms were modelled using the embedded atom method^32^. Nanoparticles, with 5.9 nm cores, were prepared as in ref. 33. The gold–thiol interaction was modelled with a Morse potential with binding strength of 10 kcal mol^−1^ (∼17 kT at 300 K), which permits ligands to migrate or detach in the presence of mechanical stresses. Nanoparticle fcc superlattices with periodic boundaries were equilibrated 0.1 GPa for 3 ns before being rotated and subjected to dynamic loading. Initial superlattice length was 10.6 nm, with nearest-neighbour core-centre spacing of 7.5 nm. Dynamic compression was modelled using a Hugoniostat approach^34^ to simulate uniaxial compression.

### Data availability

The data that support the findings of this study are available from the corresponding author upon reasonable request.

## Additional information

**How to cite this article:** Li, B. *et al*. Superfast assembly and synthesis of gold nanostructures using nanosecond low-temperature compression via magnetic pulsed power. *Nat. Commun.* **8,** 14778 doi: 10.1038/ncomms14778 (2017).

**Publisher's note**: Springer Nature remains neutral with regard to jurisdictional claims in published maps and institutional affiliations.

## Supplementary Material

Supplementary Information Supplementary Figures.
