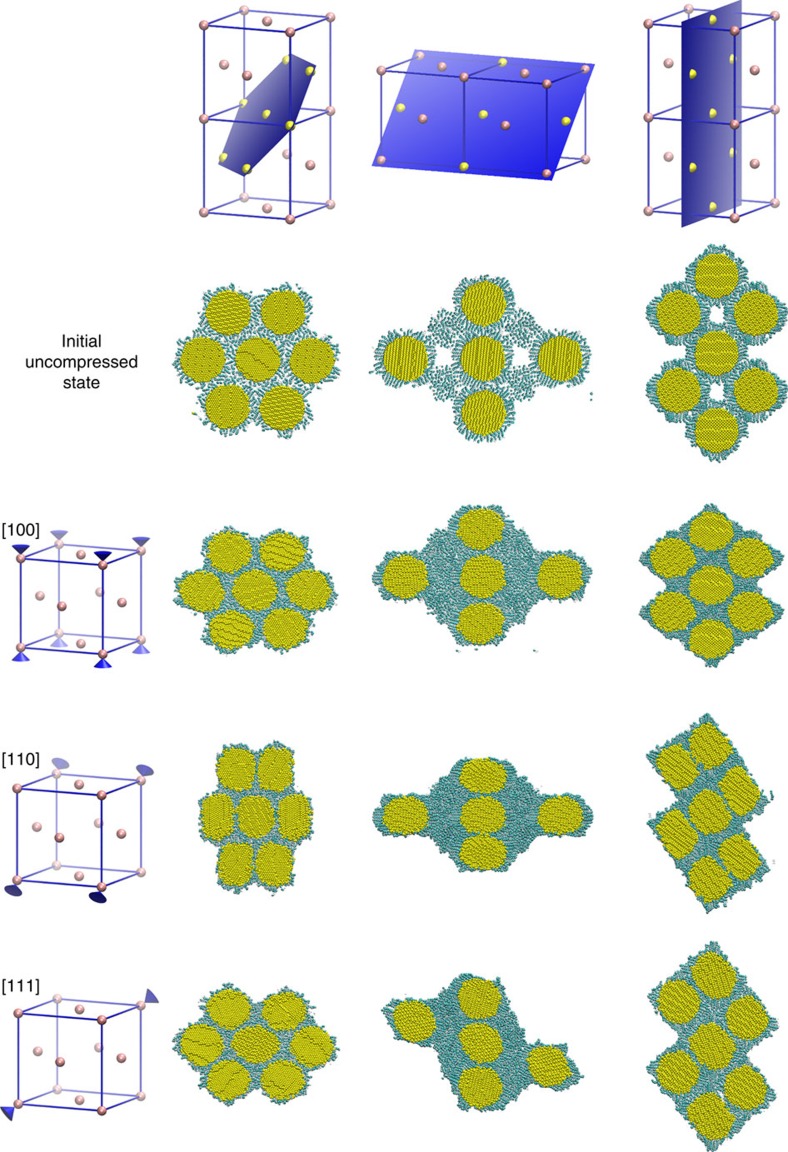# Corrigendum: Superfast assembly and synthesis of gold nanostructures using nanosecond low-temperature compression via magnetic pulsed power

**DOI:** 10.1038/ncomms15574

**Published:** 2017-05-24

**Authors:** Binsong Li, Kaifu Bian, J. Matthew D. Lane, K. Michael Salerno, Gary S. Grest, Tommy Ao, Randy Hickman, Jack Wise, Zhongwu Wang, Hongyou Fan

Nature Communications **8**: Article number: 14778; DOI: 10.1038/ncomms14778 (2017); Published: 03 16 2017; Updated: 05 24 2017

In Fig. 5 of this Article, the image in the centre column for [111] incorrectly replicates the image above. The correct version of Fig. 5 appears below as Fig. 1.

## Figures and Tables

**Figure 1:**