# Supplementary material for: Superfast assembly and synthesis of gold nanostructures using nanosecond low-temperature compression via magnetic pulsed power
Source: Nat Commun. 2017 Mar 16;8:14778. doi: 10.1038/ncomms14778 (PMC5357312; doi:10.1038/ncomms14778)
Supplement: Supplementary Information — Supplementary Figures. [file ncomms14778-s1.pdf]

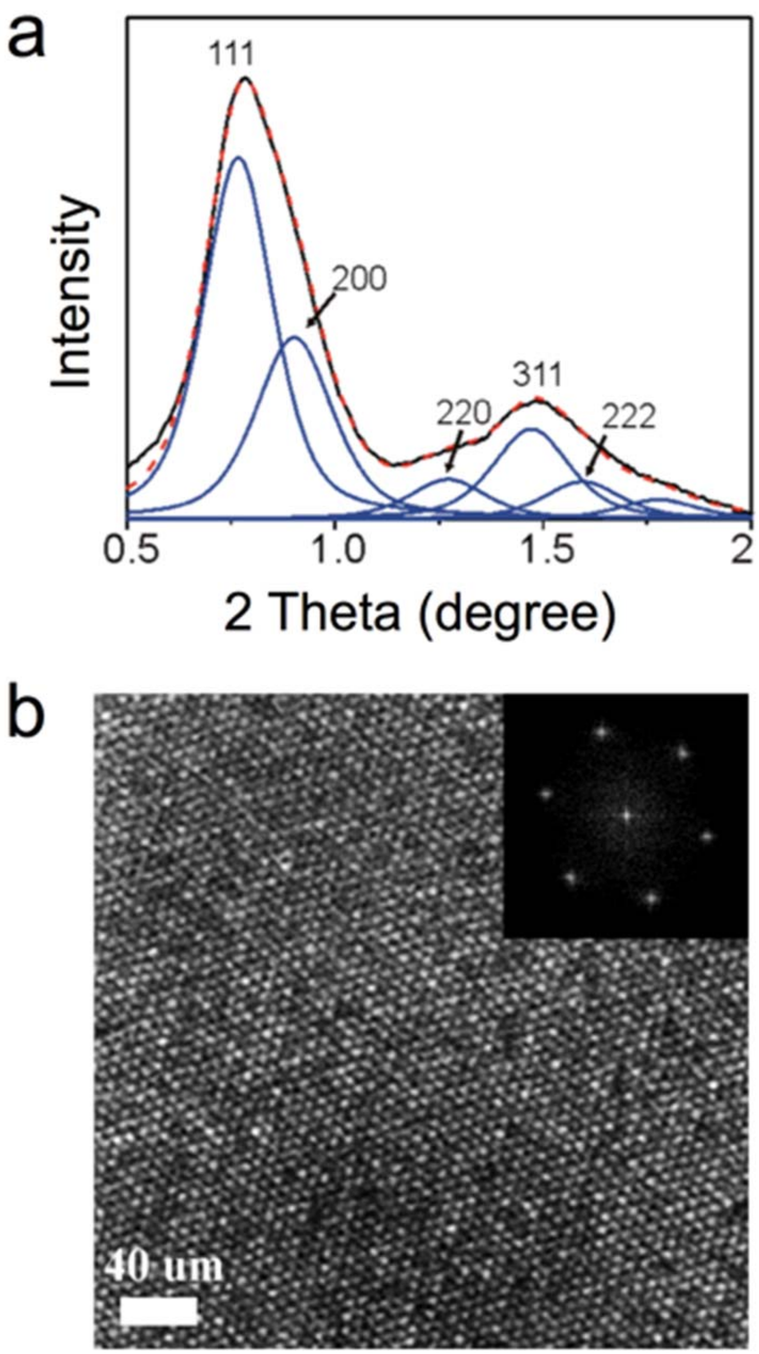

Supplementary Figure 1: Structure of the starting materials: self-assembled spherical gold nanoparticle films. (a) X-ray diffraction (XRD) spectra of a representative gold nanoparticle film. The black lines are obtained by directly integrating the collected XRD images using the Fit2D software. The red-dashed lines are the fitting curves through fcc symmetry. The blue solid curves present the peak positions of each plane. (b) SEM images of the film from top view. The insert is the FFT patterns of the periodic arrays shown in the SEM image.

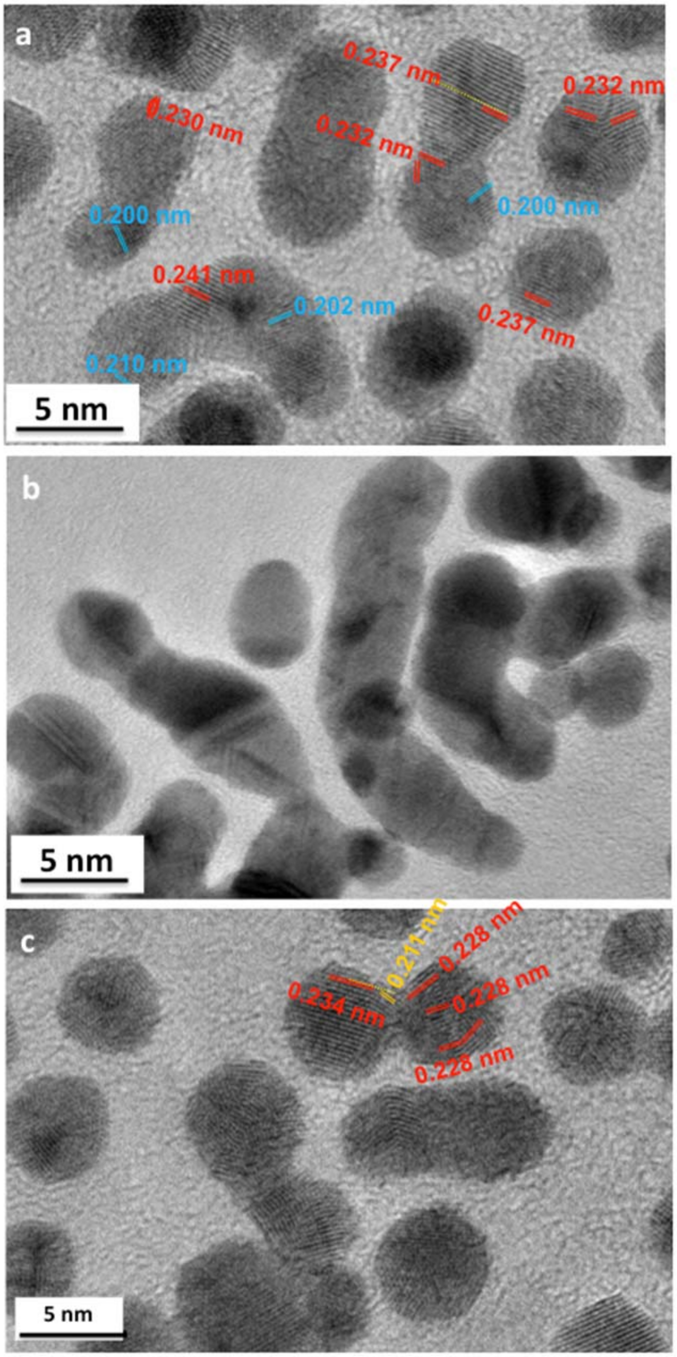

Supplementary Figure 2: TEM images of gold nanocrystal dimers, trimers, and nanorods coalesced under dynamic compression. All atomic gold lattices from these coalesced gold nanocrystals are face center cubic (111) (red lines) and (200) (blue lines). A neck is formed between two coalesced nanocrystals, which makes it easier to locate the original nanoparticles and their interfaces. The nanoparticles tend to coalesce with same faces (111 to 111, 200 to 200), where 111/111 is the major one. Distortion often was found near the interfaces of two coalesced nanocrystals, showing (1) a bending of the crystal planes up to 23 degree (c); (2) smaller crystal lattice between two coalesced nanocrystals. These indicate possible existence of residual stress.

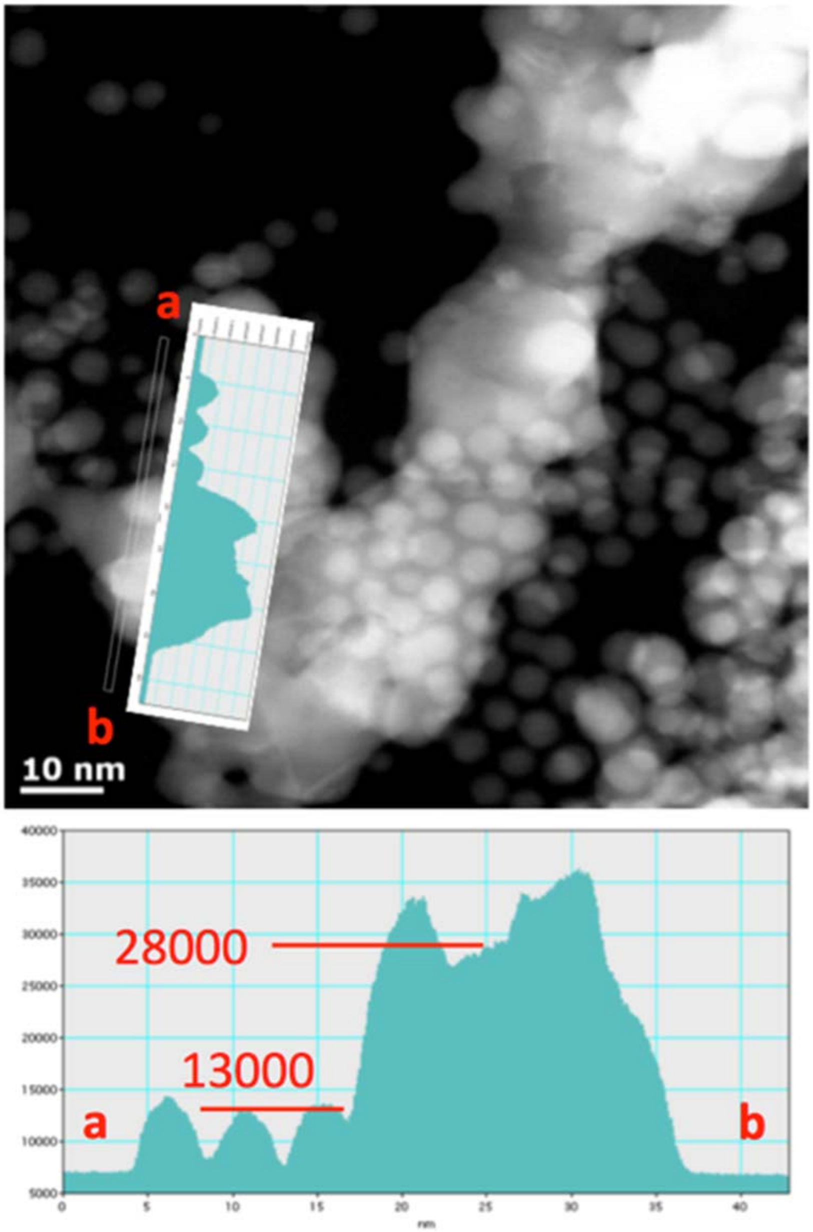

Supplementary Figure 3: Scanning TEM image of the resulted gold nanosheets with residual gold nanocrystals. Bottom: Brightness along the rectangle shape from a to b. We estimate the thickness of the gold nanosheets using nanoparticles (diameter of 6 nm) as a reference. The height profile (brightness) of the nanosheets (28000-35000) were 2-4 times as that of gold nanoparticles (8000-13000), therefore the thickness of the sheets should be 2-4 times of the diameter of the gold nanoparticles, which is about 12-24 nm.

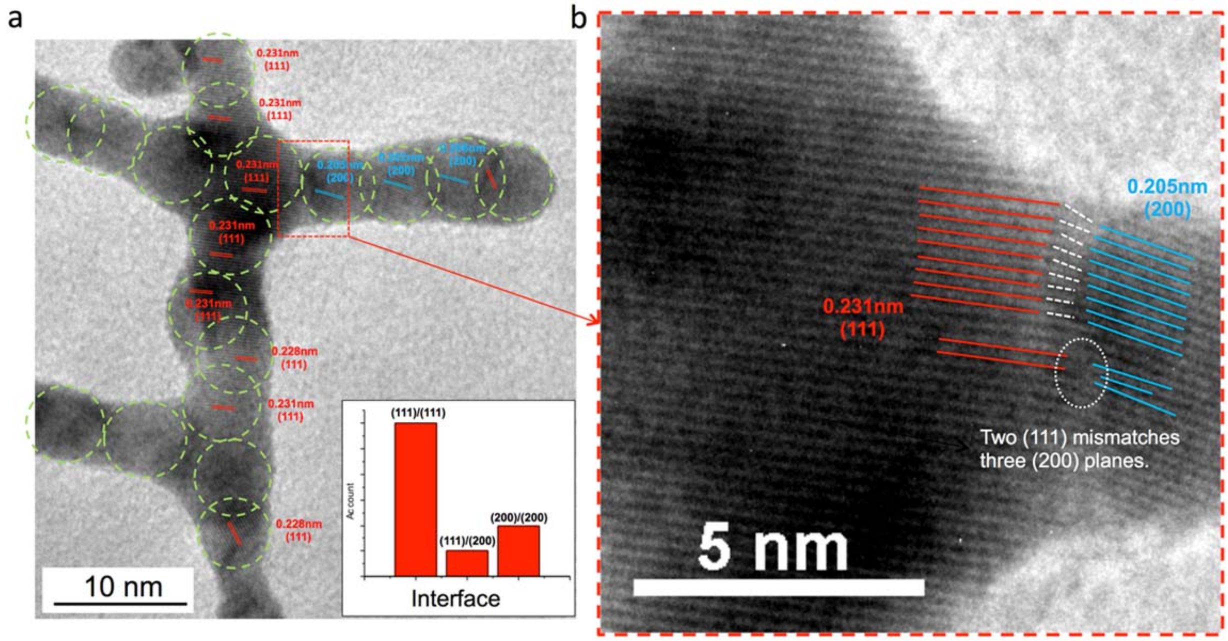

Supplementary Figure 4: High resolution TEM images of gold architectures coalesced through different planes. (a) TEM image shows interconnected gold nanocrystals highlighted by green dash circles. Statistic analyses (inset) shows gold nanocrystal coalescence occurs through different interfaces. TEM image (b) show an example highlighted in the red box in image (a) with an interface of (111) and (200) planes. The atomic crystal structure between two domains is amorphous due to the mismatch of the d spacing of (111) plane (d=0.231nm) and (200) plane (d=0.205nm).
